# Comparative efficacy of platelet-rich plasma applied in myringoplasty: A systematic review and meta-analysis

**DOI:** 10.1371/journal.pone.0245968

**Published:** 2021-01-25

**Authors:** Juntao Huang, Yunbin Shi, Linrong Wu, Cuiting Lv, Yi Hu, Yi Shen

**Affiliations:** 1 Department of Otolaryngology Head and Neck Surgery, Ningbo Medical Center of Lihuili Hospital, The Affiliated Hospital of Ningbo University, Ningbo, Zhejiang, China; 2 School of Medicine, Ningbo University, Ningbo, Zhejiang, China; Zagazig University, EGYPT

## Abstract

**Background:**

Tympanic membrane (TM) perforation is quite common in the clinical setting. Chronic TM perforations require surgical treatments such as myringoplasty. Currently, platelet-rich plasma (PRP) is a novel, effective substance that is increasingly utilized for TM perforation repair. This study aims to evaluate the effectiveness of PRP in the application of TM perforation repair.

**Methods:**

A systematic search was conducted to screen the Medline, Embase, Cochrane, Scopus and Web of Science databases up to July 2020. Studies were identified in accordance with the selection criteria by two coauthors independently. Data regarding the healing and hearing outcomes were pooled and analyzed via Review Manager version 5.3 and STATA version 12.0 software. Odds ratio (OR) was utilized to compare the closure rate. Furthermore, the results of hearing improvements and incidence of complications were also compared to evaluate the effectiveness of PRP.

**Results:**

A total of eight studies with 455 participants were eligible according to the selection criteria. Compared to conventional surgery, the OR of closure was 2.70 (95% CI: 1.27 to 5.76, *P* = 0.01, *I*^2^ = 0%) in randomized controlled trial (RCT) subgroup and 6.18 (95% CI: 2.22 to 17.25, *P* = 0.0005, *I*^*2*^ = 0) in non-RCT subgroup. The overall OR of closure was 3.69 (95% CI: 2.02 to 6.74, *P*<0.0001, *I*^2^ = 0%), suggesting a significant effect on the healing of TM perforation. Between preoperative and postoperative hearing results, there is no statistical difference between the PRP and the control groups. Additionally, the use of PRP resulted in a lower incidence of complication than the use of conventional approaches.

**Conclusion:**

The application of PRP during the TM surgeries can enhance the closure rate, provide similar hearing improvements and decrease the incidence of postoperative complications. Given these advantages, PRP can be considered an effective treatment for TM regeneration.

## Introduction

Tympanic membrane (TM) perforation represents a common problem in otology clinics. TM perforations are typically classified according to the duration of the perforation. Acute perforation tends to heal spontaneously [1], and when the disease shows a chronic progression, surgery is usually required [2]. Tympanoplasty with autografts (e.g., temporalis fascia) is the gold standard surgical treatment for TM perforation and achieves high success rates according to a previous study [3]. However, an ideal repair material has yet to be identified because of various limitations [4,5]. Patients may suffer from reperforation, middle ear infections or other complications after surgeries. Thus, it is essential to explore options to improve the efficacy of repair.

With the development of tissue engineering, various bioactive molecules have been applied during the regeneration progress with encouraging results [4–6]. Among them, platelet-rich plasma (PRP) functions as a surgical sealant and haemostatic agent, which effectively accelerated TM healing. Based on previous evidence, PRP contains many growth factors (e.g., platelet-derived growth factor, PDGF), accelerating the regeneration of endothelial tissue and enhancing tissue healing. [7–16] Although the majority of human trials have reported promising benefits [8–15], the completed closure rate varies across studies, and the outcomes of both healing and hearing improvements require further evaluation. To the best of our knowledge, no systematic analysis has been performed to compare the efficacy of PRP in TM regeneration surgeries.

Hence, this comprehensive study aims to assess the effectiveness of PRP for TM repair by evaluating the current clinical evidence via a meta-analysis.

## Materials and methods

**Fig 1:**
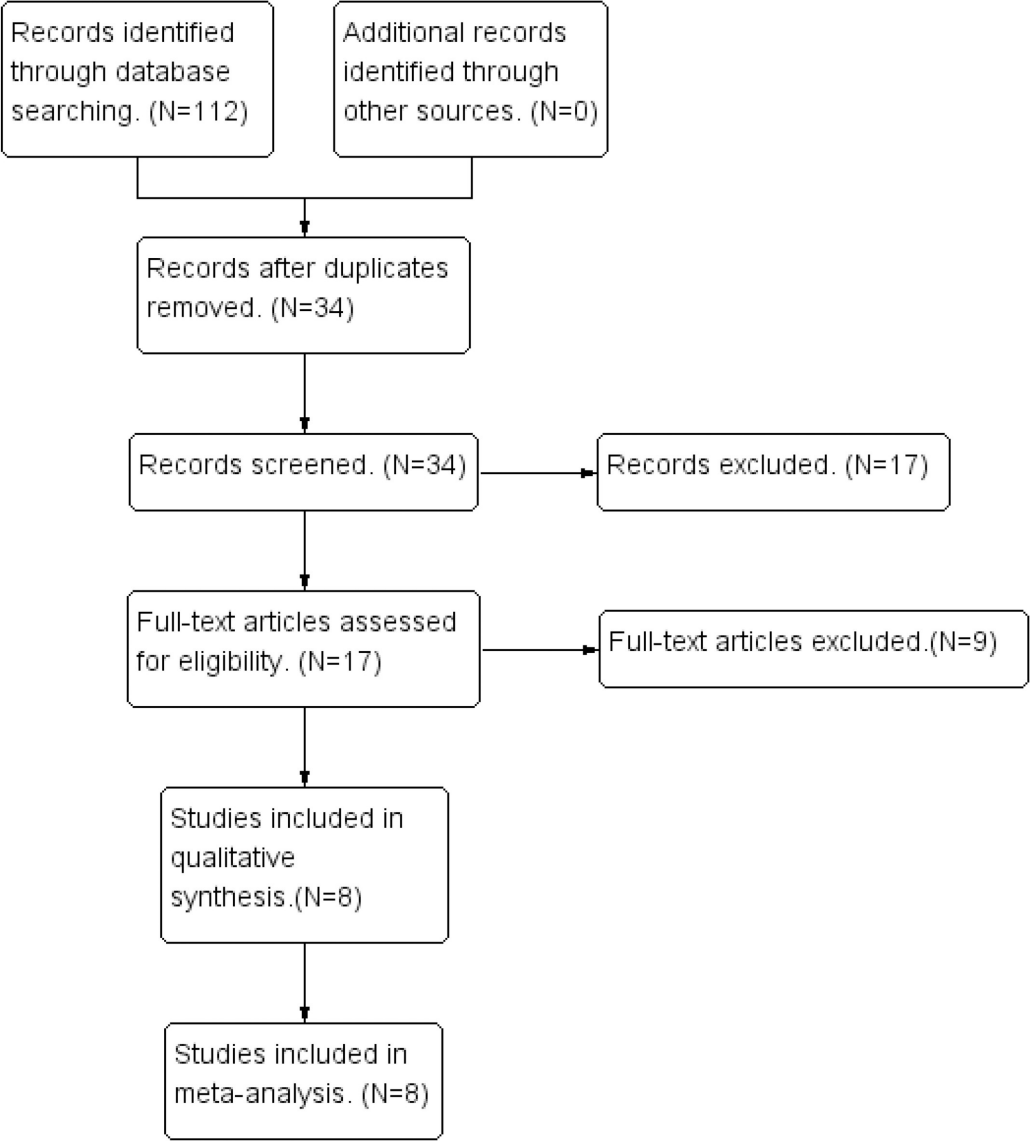
Flow diagram of the search process.

We performed this study in accordance with the Preferred Reporting Items for Systematic Reviews and Meta-Analyses (PRISMA) guidelines [17] (**Fig 1**).

### Search strategy

An online database search was conducted by using the Medline (via PubMed), Embase, Cochrane, Scopus and Web of Science databases from their inception up to July 31, 2020. Two authors (J H. and B S.) independently performed the database search, identified the studies, evaluated the quality and extracted the data from the included studies. A third author (Y S.) was consulted when any disagreement was presented between these two authors. Studies were identified by the Medical Subject Heading (MeSH) terms and text words: *1) tympanic membrane(s); membrane(s)*, *tympanic; eardrum(s); 2) tympanic membrane perforation; membrane perforation*, *tympanic; eardrum perforation; perforation*, *eardrum; tympanic membrane rupture; membrane rupture*, *tympanic; rupture*, *tympanic membrane; 3) tympanoplasty; tympanoplasties; 4) myringoplasty; myringoplasties; and 5) platelet-rich plasma; plasma*, *platelet-rich; platelet rich plasma*. The original articles were all from peer-reviewed scientific journals published in English, and duplicates were removed. The search strategy is presented in S1 File.

### Study selection

Studies were identified by screening the full-text according to the inclusion and exclusion criteria. The selection criteria were established according to the PICOS (Participants, Interventions, Comparisons, Outcomes, Studies) format [18]. The eligibility criteria were as follows: 1) patients were diagnosed with chronic TM perforation without other middle ear disorders; 2) PRP was used to assist with autografts during surgery in the experimental groups; 3) patients underwent surgery with autografts in the control groups; 4) studies that reported the outcomes of healing and hearing improvements after surgeries; and 5) observational studies (prospective or retrospective) or randomized controlled trials (RCTs). The exclusion criteria were as follows: 1) inclusion of patients with cholesteatoma, destruction of the ossicular chains or other middle ear disorders; 2) studies without control groups; and 3) case reports, reviews, letters, animal studies and in vitro studies.

### Quality assessment and statistical analysis

Subsequently, the quality of the included RCTs was judged according to the Cochrane Collaboration’s tool for assessing the risk of bias [18]. The symbols “+”, “?”, and “-” indicate a low, unclear, and high risk of bias, respectively. Similarly, retrospective studies were assessed by the Newcastle-Ottawa Scale according to the following three criteria: study selection, comparability of cohort, and assessment of outcome [19,20]. A score of six or more stars is indicative of a high-quality study [19,20] (**Table 1**). Data were independently extracted by two coauthors and included the authors, publication year, number of participants, follow-up time, size of perforation, intervention, closure rate, hearing results and postoperative complications. Comparison of closure rates were expressed as odds ratios (ORs) to evaluate the effectiveness via the Review Manager version 5.3 software (The Nordic Cochrane Centre, Copenhagen, Denmark). Moreover, a subgroup analysis was performed due to differences in study design. In addition, the *I*^*2*^ statistic was applied to assess the heterogeneity, with values of 25%, 50%, and 75% indicating low, moderate and high heterogeneity, respectively [18]. Sensitivity analysis was performed via STATA version 12 software, and publication bias was assessed via a funnel plot test [21]. The trim-and-fill method [22], was used if necessary to revise the results when publication bias was present. All outcomes were considered statistically significant when the *P* value was less than 0.05.

**Table 1:** Quality assessment for the included studies.

Study	Random sequence generation	Allocation concealment	Blinding of participants	Personnel and outcome assessors	Incomplete outcome data		Selective outcome reporting	Other sources of bias	
El-Anwar et al	+	?	?	?	+		+	?	
Anwar et al	-	?	?	+	?		?	-	
Yadav et al	+	+	+	?	+		+	?	
Mandour et al	+	+	?	+	?		+	+	
Taneja	-	+	?	+	+		?	-	
+: Low risk;?: unclear risk; −: high risk.									
Study	Representativeness of Exposed Cohort	Selection of the Nonexposed Cohort	Ascertainment of Exposure	Outcome of Interest Not Present at Start of Study	Comparability	Assessment of Outcome	Adequacy of Duration of Followup	Adequacy of Completeness of Followup	Score
Fawzy et al	*	*	*	*	*	*	*	0	7

Study	Case Definition Adequate	Representativeness of Cases	Selection of Controls	Definition of Controls	Comparability	Ascertainment of Exposure	Method of Ascertainment	Nonresponse Rate	Score
Ersozlu et al	*	*	*	*	*	*	*	0	7
Fouad et al	*	*	*	*	*	*	*	0	7

* indicates One star; **, Two stars.

## Results

### Characteristics of the included studies

Of the 34 nonrepetitive studies identified, eight studies [8–15] including 455 participants with different sizes of TM perforations were eligible for final selection after screening the full texts. These included studies published from 2015 to July 2020 and consisted of five RCTs [9,10,12–14], one prospective study [15], and two retrospective studies [8,11]. Seven of the included studies [8–10,12–15] compared the efficacy of PRP with conventional tympanoplasty or myringoplasty. Among the seven studies in which the experimental groups received PRP three involved fat graft myringoplasty (FGM) [8,9,14], one used conchal perichondrium myringoplasty (PM) [13], and three involved temporalis fascia tympanoplasty (TFT) [10,12,15]. Among the control groups, two group underwent FGM [8,14], and one group underwent PM [13]. The other four groups of patients underwent tympanoplasty surgery [9,10,12,15]. The remaining study reported the outcomes of three treatment arms [11], including PRP combined with FGM, hyaluronic acid (HA) with FGM and FGM alone. Within the studies, the follow-up time ranged 3 to 12 months. The final outcomes were determined using data that were carried forward rather than observations based on an analysis of only those who completed the study. The characteristics and statistics of the selected studies are summarized in **Table 2**.

**Table 2:** Characteristics of studies included in the meta-analysis.

Study	Year	Study Design	Cause of TMP	Follow-up(mo)	Intervention		No. of Participant(n)		Closure Rate(%)	
					Experimental Group	Control Group	Experimental Group	Control Group	Experimental Group	Control Group
Ersozlu et al [8]	2020	Retrospective	Chronic	12	PRP+M	M	32	31	100	83.9
Taneja [12]	2020	RCT	Chronic	6	PRP+T	T	41	41	95.1	85.3
Anwar et al [14]	2020	RCT	Chronic	3	PRP+T	T	35	35	88.6	77.1
Mandour et al [9]	2019	RCT	Chronic	3	PRP+M	T	25	25	88	92
Yadav et al [10]	2018	RCT	Chronic	3	PRP+T	T	20	20	95	85
Fawzy et al [15]	2018	Prospective	Chronic	6	PRP+M	M	20	20	90	55
Fouad et al [11]	2018	Retrospective	Chronic	6	PRP+M	M	21	25	85.7	60
					HA+M		23		87.0	
El-Anwar et al [13]	2015	RCT	Chronic	6	PRP+M	M	32	32	100	81.3

Abbreviations: RCT: Randomized controlled trials; PRP: Platelet-rich plasma; HA: Hyaluronic acid; M: Myringoplasty; T: Tympanoplasty.

### Closure rates

**Fig 2:**
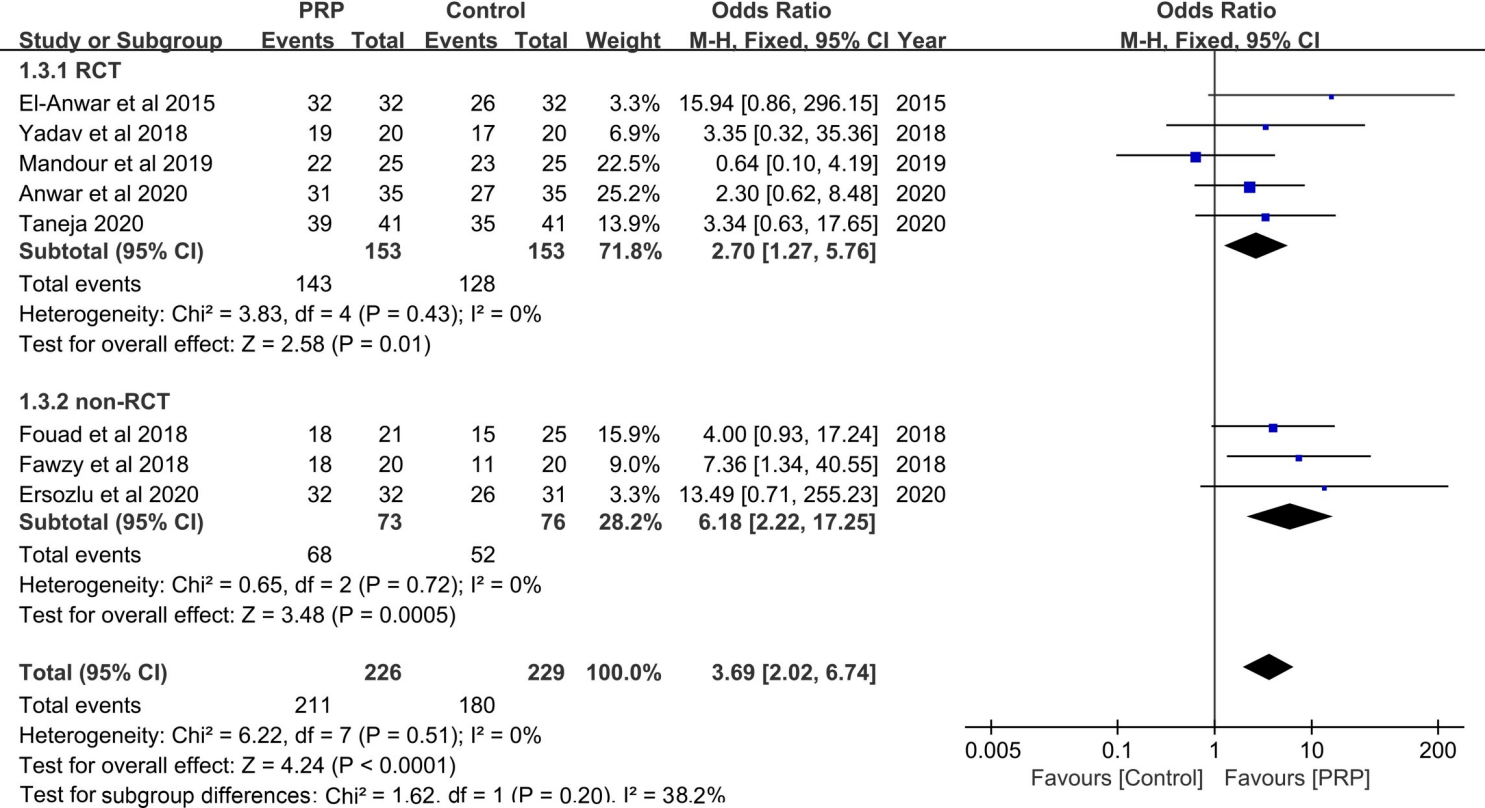
Forest plot of closure rate between PRP and control group. PRP: platelet-rich plasma; RCT: randomized controlled trial.

All of the included studies [8–15] reported the completed closure rate after surgery. The closure rate ranged from 85.7% to 100% (median 92.5%) in the PRP treatment groups and from 55% to 92% (median 82.6%) in the control groups. The overall average closure rate was 93.4% (211/226) in the PRP groups and 78.6% (180/229) in the control groups. Analysis of the closure rates revealed an OR of 2.70 (95% CI: 1.27 to 5.76, *P* = 0.01, *I*^2^ = 0%) in the RCT subgroup and 6.18 (95% CI: 2.22 to 17.25, *P* = 0.0005, *I*^*2*^ = 0) in the non-RCT subgroup. The overall OR of closure was 3.69 (95% CI: 2.02 to 6.74, *P*<0.0001, *I*^2^ = 0%), which indicates a significant enhancement of TM rupture closure with the application of PRP (**Fig 2**). These outcome results coincide with those of most previous studies, showing that PRP has a positive effect on the regeneration of TM perforation.

**Fig 3:**
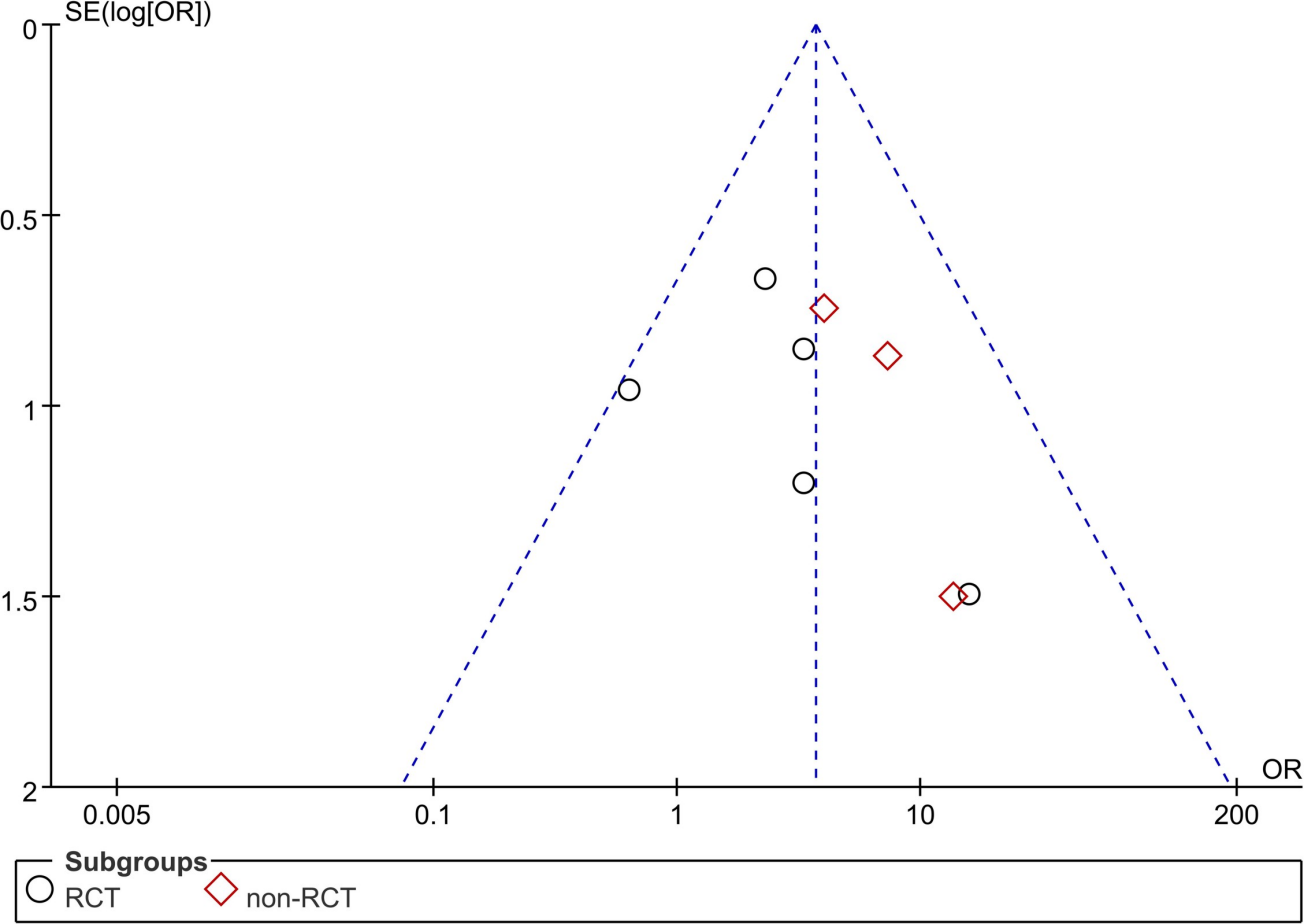
Funnel plot of closure rate between PRP and control group. PRP: platelet-rich plasma; RCT: randomized controlled trial.

A sensitivity analysis was performed, which did not change the results of this meta-analysis, indicating the results can be considered consistent and reliable. However, Begg’s test did not be performed to reflect the publication bias because the number of included studies was less than ten. The included studies were distributed equally at the two sides of the plot, which qualitatively indicates no presence of publication bias in our analysis (**Fig 3**).

### Hearing improvements

Data regarding hearing results extracted from the included studies are summarized in **Table 3** and were described in studies as preoperative and postoperative air-bone gaps (ABG) or ABG gains measured as the preoperative ABG minus the postoperative ABG at the average of frequencies. A functional success was defined as a postoperative ABG gain greater than 10 dB. All included studies [8–15] indicated the preoperative and postoperative hearing results, and four studies [10,12,13,14] reported success rate of auditory function reconstruction.

**Table 3:** Hearing results of included studies.

Study	Year	Treatment	Preoperative(dB)	Postoperative(dB)	Average Gains(dB)	ABG Gains > 10dB(n,%)	Frequencies(kHz)
Ersozlu et al [8]	2020	PRP+M	25±3.85^a^	11.5±6.84	10.3±6.74	NA	0.5,1,2,4
		M	24.3±5.65	14.81±6.94	7.23±6.72	NA	
Taneja [12]	2020	PRP+T	NA	NA	NA	32(78.0)	NA
		T	NA	NA	NA	19(46.3)	
Anwar et al [14]	2020	PRP+T	NA	NA	NA	31(88.6)	NA
		T	NA	NA	NA	27(77.1)	
Yadav et al [10]	2018	PRP+T	30.78	12.16	18.62	18(90)	NA
		T	26.94	13.79	13.15*	14(70)	
Mandour et al [9]	2019	PRP+M	20.76	6.64	14.12	NA	NA
		T	22.76	7.12	15.64	NA	
Fawzy et al [15]	2018	PRP+M	23.00±4.70	16.50±6.51	NA	NA	NA
		M	23.00±4.70	16.00±6.81	NA	NA	
Fouad et al [11]	2018	PRP+M	20.1±3.5	16.3±2.7	NA	NA	0.5,1,2
		HA+M	20.7±3.8	17.1±3.9	NA	NA	
		M	20.8±3.6	18.4±3.2	NA	NA	
El-Anwar et al [13]	2015	PRP+M	23±3.83	12.5±7.64	10.5±6.97	21(65.6)	NA
		M	22.34±8.33	14.91±7.23	7.43±7.7	13(40.6)	

^a^Values are presented as mean ± SD.

^b^Values are presented as mean.

*: Significant statistical differences between experimental groups and control groups.

Average hearing gains after surgery ranged from 10.3 to 18.62 dB in the PRP treatment groups and 7.23 to 15.64 dB in the control groups [10,12–14]. Moreover, the effective improvement rates were 65.6–90% in the PRP groups and 40.6–77.1% in the control groups [10,12–14]. All of the above results regarding hearing improvements indicate that the use of PRP with autografts may contribute to satisfactory hearing restoration in the treatment of simple chronic TM perforations. However, despite hearing improvements between the preoperative and postoperative conditions, no significant difference was observed between the PRP and control groups. After reviewing these studies, a further comparison of hearing results was not performed because data were reported for different frequencies.

### Complications during the follow-up time

Despite the lack of data exploring the incidence of complications, some patients reported complications in the included studies [8,10,13]. Yadav et al. [10] reported one and three cases of graft rejections in the PRP and control groups, respectively. Additionally, residual perforation was identified in patients who experienced repair failure within the first four postoperative months, which was observed by Ersozlu et al. [8] Moreover, in the study by El-Anwar et al. [13], four patients experienced postoperative otorrhea caused by middle ear infection in addition to residual perforation in the group without PRP treatment. Nearly all of the complications occurred in patients who experienced perforation repair failure.

## Discussion

TM perforation, if left untreated, can cause relapsing infections in the middle ear, hearing loss and otogenic complications [5,23]. Tympanoplasty is considered a relatively useful otolaryngological surgical procedure to close ruptures [24]. However, tympanomeatal flap elevation may injure the middle ear ossicles, tympanic annulus, or chorda tympani [25]. An earlobe fat graft can achieve anatomic and functional success for small perforations, but the results are less satisfactory for large perforations [26–28]. Over the past decades, alternatives to resolve the limitations of conventional tympanoplasty have been widely explored [29], including the use of scaffold materials [6], bioactive molecules [5,30] and cells [31,32]. These new technologies should ideally yield more advantages than conventional tympanoplasty. Among them, PRP is reportedly effective in wound healing and present great potentiality for TM repair. Since Navarrete Alvaro et al. demonstrated the preliminary outcomes of PRP in myringoplasty in 2001 [33], it has been applied in cases of chronic perforation with autografts to achieve satisfactory closure rates [8–16]. However, most of these studies included small numbers of patients. Studies that quantitatively evaluate the effectiveness of PRP in a large number of participants are lacking. Hence, we used a systematic analysis to assess the efficacy of PRP in TM regeneration surgery.

The complete closure rate serves as the most important standard to evaluate the effectiveness of TM regeneration technology [2,34]. In our review, the average closure rate in the PRP treatment groups was 93.4%, compared to 78.6% in the control groups with conventional surgery alone. In addition, the OR (3.69) of healing demonstrated that application of PRP along with autografts achieve a higher success rate in repairing perforations than conventional tympanoplasty or myringoplasty. However, differences in the types of autografts may influence the final success rate and contribute to the heterogeneity in analysis. The heterogeneity for the total group and each subgroups was 0%. Preclinical and clinical evidence from previous studies also supports our analysis results [7–13]. The sensitivity analysis also indicated that the results comparing the closure rates using different graft materials were reliable and consistent. Furthermore, when combined with a fat graft, the closure rate in the PRP group was 85.7%, compared with a rate of 87.0% in the HA group, indicating that the use of PRP seems to exhibit similar effectiveness to application of HA during surgery.

In addition to the healing results, hearing improvements also play an important role in the evaluation of alternative strategies [35,36]. Yadav et al [10] indicated that TFT with the addition of PRP can provide greater hearing improvement. However, according to our review, most of the included studies indicated no significant differences in hearing results between the PRP and control groups [8,9,11,13]. The application of PRP seems to provide similar effectiveness in terms of hearing improvements to conventional approaches. This discrepancy in between hearing results and healing results may be due to the small number of enrolled patients. In addition to adding more studies with larger populations to obtain more reliable results, other factors also influenced the hearing results [5]. For patients with TM perforations, the outcome of hearing function for TM repair depends on factors such as graft type, surgical approach, Eustachian tube function, and the site and size of perforations [35,36]. Among the included studies, patients had simple perforations without other serious middle ear disorders, especially ossicular chain interruption, which may possibly explain the lack of differences between the two groups in terms of hearing improvements. Moreover, all of the hearing results were reported during a short-term follow-up [8–13], and long-term hearing results after the surgeries still remain to be explored.

Additionally, application of PRP also was associated with a low incidence of complications after surgery. Across our review, only one case of graft rejection was reported when PRP was used in TM surgery [10]. The case of graft rejection occurred in a patient who experienced perforation repair failure. For patients who had complete TM healing, no grafts were lateralized or displaced into the middle ear, and no retraction pockets or postoperative complications, such as sensorineural hearing loss, tympanosclerosis or thin atrophic areas, occurred during the follow-up period [13]. Furthermore, referring to the four cases of postoperative infection in the control group without PRP indicated by El-Anwar et al. [13], PRP may have a bactericidal action due to the presence of white blood cells (WBCs) [37].

The mechanism of PRP in TM regeneration has been explored and reported in animal models and human trials [38–40]. PRP is believed to play a role in promoting healing and preventing dehydration of the perforation margins [9]. As an autologous bioactive molecule, PRP serves as a growth factor agonist and has both chemotactic and mitogenic properties [41,42]. After the patients’ own venous blood is separated into three layers via centrifugation, PRP is extracted from the middle layer and contains platelets and WBCs [15]. PRP contains fibrin, fibronectin and autologous growth factors to promote cell proliferation, migration, and angiogenesis [43]. It can also be activated by PDGF and can release PDGF to promote wound healing [8,40].

Although PRP is used in TM regeneration surgery, otologists use PRP for different purposes in repairing perforations. In chronic perforation treatment, PRP is always combined with autografts to repair the TM [8–15]. It is mostly prepared and inserted into the external ear canal on the outer face of the TM remnant after an autograft is used to cover the perforation. According to the included studies, several types of autografts have been applied with PRP to explore their effectiveness, including temporalis fascia [9], conchal perichondrium [13] and fat grafts [8,9,11]. Saeedi et al [14] combined Gelfoam with PRP to repair perforations, and the closure rates in the PRP group (66.7%) and the control group (25%) were both lower than those in other studies using autografts. Alhabib et al. [44] also inserted PRP directly through the perforation toward the middle ear rather than in combination with autografts to repair perforations. This study was aborted due to a low success rate of 18.2% [2/11]. The only two successful cases in the PRP group also had a tendency to retract medially because of a weak membrane. These results suggested that surgeons should consider the rigidity and adsorption of transplant materials when using PRP to repair TM perforation. Although the optimal matching autograft has yet to be identified due to a lack of relevant research, a similar study examining the application of HA with FGM [28] may indirectly reveal the feasibility of using adipose tissue with PRP. Compared to other conventional surgical approaches (e.g., TFT), FGM is performed as a minimally invasive approach without elevation of the tympanomeatal flap and can be performed under local anesthesia [26]. Adipose tissue can produce numerous proinflammatory and proangiogenic proteins, resulting in enhanced revascularization due to its increased secretory activity [45,46]. However, the success rate of FGM is associated with the perforation site and size [26]. For patients with large perforations, FGM alone may not be as satisfactory as other approaches due to adipose resorption before achieving complete closure. However, with the addition of PRP, the complete closure rate is enhanced, even with large perforations [8,13]. PRP can also be inserted into the perforation directly without autografts as described by Alhabib et al. [17], while this strategy achieved success in only two cases. The outcomes of PRP monotherapy in chronic TM perforation treatments seem less successful due to the weak mechanical properties of PRP. Therefore, according to the previous studies, it seems more advisable to use the combination of PRP with FGM in the treatment of chronic TM perforation.

Furthermore, there are several advantages of the use of PRP over than other bioactive molecules (e.g., HA) in TM regeneration. The safety and low toxicity of HA have been determined [28,29,47], whereas some cases of sensorineural hearing loss in guinea pigs were reported by Antonelli and colleagues. [48] As an autologous material, PRP does not induce any undesirable tissue reactions, which can ensure safety for patients. In addition, although favorable closure rates with HA and FGM suggest the potential for cost savings, Wong et al. noted that the potential long-term cost savings may be offset by the higher upfront costs of HA ester [28]. PRP is easy, quick and inexpensive to produce and can easily be manipulated during surgical procedures [8–16]. For patients who undergo PRP therapy, the healing time of TM regeneration [49] and hospitalization duration [16] were shortened. These results are quite clinically relevant, since earlier closure reduces medical costs associated with treatment and follow-up; in addition, the need for fewer precautions to prevent water from entering the ear canal allows patients to return to social activities more quickly [5].

However, there are several limitations of our study. A major limitation is the lack of relevant studies to further assess hearing improvements and postoperative complications. Additionally, the measurement used to assess our critical outcome involved the use of various autografts when reconstructing the TM. To make the results more consistent, further original studies should be conducted with a larger population. However, based on the similarity to outcomes presented in previously published studies, the outcomes of this analysis can be considered reliable. Only studies published in English fulfilled the inclusion criteria and were included in the study, which may cause selection bias. Further studies should be focused on patients with other middle ear disorders as well as comparison of PRP with other bioactive molecules.

## Conclusion

According to our meta-analysis, the application of PRP during TM surgeries can enhance the closure rate, provide potentially greater hearing improvements and decrease the incidence of postoperative complications. Given these advantages, PRP can be considered an effective material in TM regeneration.

## Supporting information

S1 File PRISMA 2009 checklist. (DOC)

S2 File PRISMA 2009 flow diagram. (DOC)

S3 File (ZIP)

S4 File (DOC)
