# Supplementary material for: Comparative efficacy of platelet-rich plasma applied in myringoplasty: A systematic review and meta-analysis
Source: PLoS One. 2021 Jan 25;16(1):e0245968. doi: 10.1371/journal.pone.0245968 (PMC7833258; doi:10.1371/journal.pone.0245968)
Supplement: S2 File — (DOC) [file pone.0245968.s002.doc]

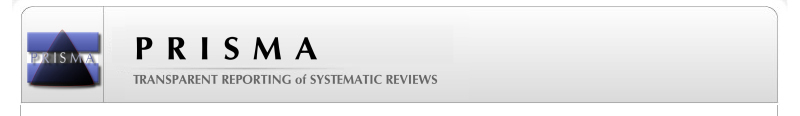
**PRISMA 2009 Flow Diagram**

**Screening**

**Included**

**Eligibility**

**Identification**

Records identified through database searching
(n =112)

Additional records identified through other sources
(n =0)

Records after duplicates removed
(n =34)

Records screened
(n =34)

Records excluded
(n =17)

Full-text articles assessed for eligibility
(n =17)

Full-text articles excluded, with reasons
(n =9)

Studies included in qualitative synthesis
(n =8)

Studies included in quantitative synthesis (meta-analysis)
(n =8)
