# Supplementary material for: Comparative efficacy of platelet-rich plasma applied in myringoplasty: A systematic review and meta-analysis
Source: PLoS One. 2021 Jan 25;16(1):e0245968. doi: 10.1371/journal.pone.0245968 (PMC7833258; doi:10.1371/journal.pone.0245968)
Supplement: S4 File — (DOC) [file pone.0245968.s004.doc]

1. Ersozlu T, Gultekin E. A Comparison of the Autologous Platelet-Rich Plasma Gel Fat Graft Myringoplasty and the Fat Graft Myringoplasty for the Closure of Different Sizes of Tympanic Membrane Perforations. Ear Nose Throat J. 2020; 99: 331-6. https://doi.org/10.1177/0145561319900388 PMID: 31928083
2. Mandour MF, Elsheikh MN, Khalil MF. Platelet-Rich Plasma Fat Graft versus Cartilage Perichondrium for Repair of Medium-Size Tympanic Membrane Perforations. Otolaryngol Head Neck Surg, 2019; 160: 116-21. https://doi.org/10.1177/0194599818789146 PMID: 30037309
3. Yadav SPS, Malik JS, Malik P et al. Studying the result of underlay myringoplasty using platelet-rich plasma. J Laryngol Otol, 2018; 132: 990-4. https://doi.org/10.1017/s0022215118001846 PMID: 30370872
4. Fouad YA, Abdelhady M, El-Anwar M et al. Topical platelet rich plasma versus hyaluronic acid during fat graft myringoplasty. Am J Otolaryngol, 2018; 39: 741-5. https://doi.org/10.1016/j.amjoto.2018.08.004 PMID: 30173940
5. Taneja MK. Role of Platelet Rich Plasma in Tympanoplasty. Indian J Otolaryngol Head Neck Surg; 2020, 72: 247-50. https://doi.org/10.1007/s12070-020-01815-y PMID: 32551285 PMCID: PMC7276456
6. El-Anwar MW, El-Ahl MAS, Zidan AA et al. Topical use of autologous platelet rich plasma in myringoplasty. Auris Nasus Larynx, 2015; 42: 365-8. https://doi.org/10.1016/j.anl.2015.02.016 PMID: 25794691
7. Anwar FM; Shenoy VS; Kamath PM et al. Study on Use of Platelet-Rich Plasma in Myringoplasty. Indian Journal of Otology. 2020; 26(2): 71-4. DOI: 10.4103/indianjotol.INDIANJOTOL_103_18; WOS: 000553530800003
8. Fawzy T, Hussein M; Eid S et al. Effect of adding platelet-rich plasma to fat grafts in myringoplasty. Egyptian Journal of Otolaryngology. 2018; 34(4): 224-8 DOI: 10.4103/ejo.ejo_53_18; WOS: 000456834900003
