# Supplementary material for: Comparative efficacy of platelet-rich plasma applied in myringoplasty: A systematic review and meta-analysis
Source: PLoS One. 2021 Jan 25;16(1):e0245968. doi: 10.1371/journal.pone.0245968 (PMC7833258; doi:10.1371/journal.pone.0245968)
Supplement: S3 File — (ZIP) [file pone.0245968.s003.zip › s1/Search strategy.doc]

Medline via Pubmed

#1 "Tympanic Membrane"[Mesh] OR "Tympanic Membrane Perforation"[Mesh]

#2 "Tympanoplasty"[Mesh] OR "Myringoplasty"[Mesh]

#3 "Platelet-Rich Plasma"[Mesh]

#4 ((((((((((((Membrane, Tympanic[Title/Abstract]) OR (Membranes, Tympanic[Title/Abstract])) OR (Tympanic Membranes[Title/Abstract])) OR (Eardrum[Title/Abstract])) OR (Eardrums[Title/Abstract])) OR (Membrane Perforation, Tympanic[Title/Abstract])) OR (Eardrum Perforation[Title/Abstract])) OR (Perforation, Eardrum[Title/Abstract])) OR (Tympanic Membrane Rupture[Title/Abstract])) OR (Membrane Rupture, Tympanic[Title/Abstract])) OR (Rupture, Tympanic Membrane[Title/Abstract])) OR (Tympanoplasties[Title/Abstract])) OR (Myringoplasties[Title/Abstract])

#5 (Plasma, Platelet-Rich[Title/Abstract]) OR (Platelet Rich Plasma[Title/Abstract])

#6 #1 OR #2 OR #4

#7 #3 OR #5

#8 #6 AND #7

Cochrane Library

#1	MeSH descriptor: [Tympanic Membrane] explode all trees

#2	MeSH descriptor: [Tympanic Membrane Perforation] explode all trees

#3	MeSH descriptor: [Tympanoplasty] explode all trees

#4	MeSH descriptor: [Myringoplasty] explode all trees

#5	MeSH descriptor: [Platelet-Rich Plasma] explode all trees

#6	(tympanic membrane) OR (tympanic membrane perforation) OR (tympanoplasty) OR (myringoplasty) (Word variations have been searched)

#7	(Membrane, Tympanic):ti,ab,kw OR (Membranes, Tympanic):ti,ab,kw OR (Tympanic Membranes):ti,ab,kw OR (Eardrum):ti,ab,kw OR (Eardrums):ti,ab,kw (Word variations have been searched)

#8	(Membrane Perforation, Tympanic):ti,ab,kw OR (Eardrum Perforation):ti,ab,kw OR (Perforation, Eardrum):ti,ab,kw OR (Tympanic Membrane Rupture):ti,ab,kw OR (Membrane Rupture, Tympanic):ti,ab,kw (Word variations have been searched)

#9	(Rupture, Tympanic Membrane):ti,ab,kw OR (Tympanoplasties):ti,ab,kw OR (Myringoplasties):ti,ab,kw (Word variations have been searched)

#10	(Plasma, Platelet-Rich):ti,ab,kw OR (Platelet Rich Plasma):ti,ab,kw

#11	#1 OR #2 OR #3 OR #4 OR #6 OR #7 OR #8 OR #9

#12	#5 OR #10

#13	#11 AND #12

Embase

#1 'eardrum'/exp OR 'eardrum perforation'/exp OR 'tympanoplasty'/exp OR 'myringoplasty'/exp OR eardrum OR (eardrum AND perforation) OR tympanoplasty OR myringoplasty OR 'tympanic membrane':ab,ti OR 'membrane, tympanic':ab,ti OR 'membranes, tympanic':ab,ti OR 'tympanic membranes':ab,ti OR eardrums:ab,ti OR 'membrane perforation, tympanic':ab,ti OR 'tympanic membrane perforation':ab,ti OR 'perforation, eardrum':ab,ti OR 'tympanic membrane rupture':ab,ti OR 'membrane rupture, tympanic':ab,ti OR 'rupture, tympanic membrane':ab,ti

#2 'thrombocyte rich plasma'/exp OR (thrombocyte AND rich AND plasma) OR 'plasma, platelet-rich':ab,ti OR 'platelet-rich plasma':ab,ti OR 'platelet rich plasma':ab,ti

#3 #1 AND #2

Web of Science

#1 (TS=platelet-rich plasma) OR (ALL=((Plasma Platelet-Rich) OR (Platelet Rich Plasma) OR (PRP))

#2 (TS= tympanic membrane) OR (TS= tympanic membrane perforation) OR (TS=(Tympanoplasty OR Myringoplasty) OR (ALL=((Membrane Tympanic) OR (Membranes Tympanic) OR (Tympanic Membranes) OR (Eardrum) OR (Eardrums) OR (Membrane Perforation Tympanic) OR (Eardrum Perforation) OR (Perforation, Eardrum) OR (Tympanic Membrane Rupture) OR (Membrane Rupture Tympanic) OR (Rupture Tympanic Membrane) OR (Tympanoplasties) OR (Myringoplasties))

#3 #1 AND #2

Scopus

(TITLE-ABS-KEY((Membrane Tympanic) OR (Membranes Tympanic) OR (Tympanic Membranes) OR (Eardrum) OR (Eardrums) OR (Membrane Perforation Tympanic) OR (Eardrum Perforation) OR (Perforation Eardrum) OR (Tympanic Membrane Rupture) OR (Membrane Rupture Tympanic) OR (Rupture Tympanic Membrane) OR (Tympanoplasties) OR (Myringoplasties) OR (tympanic membrane) OR (tympanic membrane perforation) OR (tympanoplasty) OR (myringoplasty))) AND (TITLE-ABS-KEY((Plasma Platelet-Rich) OR (Platelet Rich Plasma) OR (PRP) OR (platelet-rich plasma)))
